# Editorial: Rising stars in neurotoxicology: 2021

**DOI:** 10.3389/ftox.2023.1172417

**Published:** 2023-03-10

**Authors:** Katharina Koch

**Affiliations:** ^1^ IUF—Leibniz Research Institute for Environmental Medicine, Duesseldorf, Germany; ^2^ DNTOX GmbH, Duesseldorf, Germany

**Keywords:** neurotoxicology, NAM, TBI-traumatic brain injury, organophoshpates, PFOA (perfluorooctanoate), artificial intelligence, fentanyl, brain barrier

Neurotoxicology in the 21st century increasingly focuses on integrated approaches to testing and assessment (IATA) that integrate epidemiological, *in vivo*, *in vitro*, and *in silico* data into toxicology evaluation for regulatory purposes (Krewski et al., 2020). Thus, current research has a strong focus on elucidating the mechanistic underpinnings of pathologies associated with neurotoxicant exposure. Moreover, novel translational bioinformatics and chemo-informatics approaches, such as machine learning and artificial intelligence (AI) become increasingly important in predicting neurotoxicity (Aschner et al., 2022). This article collection covers both *in vivo* and *in vitro* studies performed in different species and features review articles elucidating the importance of closing data gaps regarding the pathomechanisms of neurotoxicants and other exogenous noxae harming the brain. Given the peculiarities of the developing brain and its particular vulnerability to exogenous noxae (Rice & Barone, 2000), we integrated articles differentially considering the effects of environmental neurotoxic compounds or brain injuries in a developmental stage-specific approach. This article collection shall offer a platform to emerging researchers in the field of neurotoxicology and compile a selection of studies that elucidate the broad method portfolio and progressiveness of the research field and further highlight its impact for the global population.

The first article of Lui et al. addresses the challenges of severe traumatic brain injury (TBI) management in children. Brain injuries caused by external forces are of global concern and the likelihood of sustaining a TBI is especially high for young children and adolescents, however, clinical management plans are often tailored for the treatment of adults. This article elucidates the necessity to adapt the clinical management when treating pediatric TBI patients by elaborating on TBI pathomechanisms and outlining the main physiological and anatomical differences between adults and children that impact TBI management. The article further proposes a clinical algorithm for the differential management of severe TBI in children and adults including guidelines for neuroimaging, CSF drainage, management of blood pressure, fluids, glucose and temperature, antiseizure medications and sedation and analgesia. Finally, both recovery from TBI and long-term outcomes are discussed with respect to the patient age.

The second article of Lam et al. studies consequences of sub-chronic fentanyl exposure in neuron and glial co-cultures to unravel pathomechanisms underlying the analgesic’s neurological side effects. As a frequently used opioid in pain management, fentanyl can elicit neurological side effects, however, its precise molecular effects on brain cells remain elusive. The authors studied the sub-chronic electrophysiological phenotypic and transcriptomic profile of low and high dose fentanyl exposure on multicellular primary rat cultures containing cortical neurons, astrocytes, and oligodendrocyte precursors. High dose fentanyl (10 µM) caused a decline in spike and burst rates of matured neuronal networks already 30 min post exposure. Transcriptomics revealed altered expression pattern of genes crucial for synaptic transmission, inflammation, and extracellular matrix organization upon low-dose exposure, indicating that fentanyl affects synaptic plasticity and thus neurological function at low doses.

The third contribution of Wang et al. uses convolutional neural network (CNN) models to evaluate neurotoxicity in live cell imaging. A biomarker-optimized CNN (BO-CNNs) was used to quantify signals of genetically encoded death indicators (GEDI) that mark irreversible neuronal commitment to cell death based on intracellular Ca^2+^ levels. The BO-CNN was tested to identify neuronal cell death based on GEDI signals in the cell morphology or nuclear fraction. Especially training of the BO-CNN with signals from nuclear-localized fluorescent proteins facilitated the identification of irreversible cell death, focusing on novel small punctate signals within the nucleus, a previously unidentified phenomenon associated with cell death that is difficult to distinguish by eye. The use of fluorescent nuclear morphology markers in combination with CNN-assisted image analysis can facilitate the identification of neurotoxicity in live imaging.

The fourth article of Merrill et al. studies how gestational exposure to perfluorooctanoic acid (PFOA) influences maternal and “anxiety-like” behavior in dams. Although PFOA, an environmental contaminant and known endocrine disruptor, is known to cause “anxiety-like”-behavior in adult male mice and gestationally exposed offspring by disturbing the hypothalamic-pituitary-adrenal axis, effects of PFOS exposure on maternal mental health remain elusive. The authors observed that low-dose PFOA exposure of dams correlated with altered maternal behavior. Exposed dams featured a passive nursing posture, reduced latency to first contact with the pup and reduced retrieval latency. Exposed dams further displayed increased “anxiety-like” behavior at weaning, with significantly higher mean duration in closed arms of the elevated plus maze. The study improves the understanding how the persistent environmental contaminant PFOA can affect maternal depression-associated and anxiety-like behavior.

The fifth article of Ireland et al. differentially studies the neurotoxic effects of organophosphorus pesticides (OPs) in adult and regenerating freshwater planarians. The authors evaluated morphological and behavioral effects of 7 OPs (acephate, chlorpyrifos, dichlorvos, diazinon, malathion, parathion and profenofos) in adult and regenerating planarians to discriminate adult from developmental neurotoxicity (DNT). Although OPs are known disrupters of acetylcholinesterase (AChE), the authors found that the toxicological profiles did not correlate with the level of AChE inhibition. Comparison of OP phenotypic profiles with effects of mechanistic control compounds for reported OP target (cholinergic neurotransmission, serotonin neurotransmission, endocannabinoid system, cytoskeleton, adenyl cyclase and oxidative stress) revealed that the clustering of the phenotypic profiles highly depended on the developmental stage, highlighting that OPs have different targets in the developing and adult brain.

The sixth article of Bell and O’Shaughnessy, focusses on the importance of the development and integrity of brain barriers for chemical toxicity. The developing brain is especially vulnerable to xenobiotics, since the blood-brain and the blood-cerebrospinal fluid barriers have not been fully matured. The review article provides a species comparison of the cellular and physiological makeup of the brain barrier systems also in respect of their limited functionality during brain development. Furthermore, the authors provide two case studies where they focus on how two environmental compound classes of high global relevance, perfluoroalkyl substances (PFAS) and bisphenols, penetrate barriers in adult and developing brains. With this review the authors highlight the importance of brain barriers for the toxicokinetics and associated risks of environmental xenobiotics.
